# University-industry-government relations of the Ministry of Industry and Information Technology (MIIT) universities: The perspective of the mutual information

**DOI:** 10.1371/journal.pone.0211939

**Published:** 2019-02-08

**Authors:** Rui Li, Weihua Fang

**Affiliations:** School of Public Administration, Beihang University, Beijing, China; University of California San Francisco, UNITED STATES

## Abstract

The Ministry of Industry and Information Technology (MIIT) universities are important bases for science and technology research and play a critical role in China’s National Innovation System. Based on the Web of Science (WoS), this article analyzes the statistics of paper published by MIIT universities and universities from across China including MIIT universities. The results are as follows: (1) Both the MIIT universities and universities nationwide in China have increased their international academic publications, and MIIT have shown a greater increase for the past decade. (2) In terms of U-I-G interaction, for UG relations, the *T*_*ug*_ value of MIIT universities has remained stable, while that of universities in China has become declined. For UI relations, the *T*_*ui*_ value of both MIIT universities and universities in China has shown steady growth. For UIG relations, MIIT universities have a greater synergistic effect of Triple Helix relationship than universities in China. (3) For more details in seven MIIT universities, universities elected into “Project 985”, including HIT, BUAA, BIT and NPU, have published more papers, and been more synergistic with government and industry (UIG relations) than other three universities, including NUAA, NUST and HEU. Based on the empirical results, we discuss our findings, and make certain suggestions regarding policy incentives, reasonable administrative system and U-I-G interaction mode, which is significant not only for Chinese universities but also for universities in other developing countries.

## Introduction

In the era of knowledge-based economy, innovation has been the driving force of the national development. To thrive knowledge economy through innovation in one country, a reasonable institutional framework is needed, and institutional actors of a nation are expected to develop new patterns of interaction [1]. Some normative frameworks of interactions among university, industry and government have been constructed and used by researchers to study and understand interactions among key actors in innovation systems. Traditionally, the academic discourse for national innovation system regards the relationships among universities, industry and government (UIG) as linear. This model, however, overemphasizes the attributes of individual institutions and neglects the interactive relationships among them [1, 2, 3]. The Triple Helix model, characterized as a networked overlay of interactions among UIG, makes up knowledge function in the innovation-driven society by adjusting the social governance structure [4].

One of the key concepts about the Triple Helix is the entrepreneurial university. It operates according to an interactive rather than a linear model of innovation. As the producer and transmitter of knowledge, modern universities have a substantial impact on regional economic development by collaborating with other innovation actors, including government departments and enterprises. During this process, universities can gain additional funding sources to improve the performance of research. They are referred to as “entrepreneurial universities” [4]. There have been a number of studies on entrepreneurial universities since the Triple Helix concept was introduced into China. These studies include the description about the non-linear paths of the entrepreneurial universities [5], the role of universities in fostering knowledge-intensive clusters [6], and the barriers to entrepreneurial universities’ growth [7].

These studies shared the view that the Triple Helix model is a solution for entrepreneurial universities in China. Meanwhile, as the mutual information among uncertainties in three dimensions, a Triple Helix indicator helps us to study the extent to which networks of relations among university, industry and government have developed into a synergistic configuration. This methodology has been used for the analysis of innovation systems, including sectoral, technological and regional ones [8], but few studies have been made to analyze and compare the development of universities with different models in one country. This paper not only presents MIIT universities in China, whose interaction with other key innovation actors, but also, more importantly, compares the research performance of interactive modes between the MIIT universities and all universities in China.

## Research framework: The Triple Helix model and relevant literature

The Triple Helix model evolves from two opposing standpoints, namely statist and lasses-fair model [4, 9]. In the statist model, government controls both industries and universities, and typical examples for this model include the former Soviet Union, France and China. In the laissez-fair model, university, industry and government are separate and independent from each other. This model is typically exemplified by the United States. The Triple Helix model has been widely used to analyze interaction among university, government and industry from the perspective of international comparative studies. Despite its popularity, the Triple Helix model has been criticized for paying little attention to national contexts [10, 11]. For example, these existing studies about Chinese innovation system are on the premise of the statist model. In fact, there are different interactive models among university, industry and government within Chinese innovation system based on different traditions [12]. While the government overall controls the development of industry and university, there are different interactive models among university, government and industry. The seven MIIT universities are examples as new models.

The Triple Helix model has had an extensive and profound influence on academic research and policy-making authorities since it was first proposed. A series of research results have been achieved including the evolution of regional innovation system [13], the dynamic mechanism of the Triple Helix model [14, 15], the function of universities, and the rise of entrepreneurial universities [5].

In this paper, we compare the research performance between MIIT universities and universities nationwide in China. The reason we choose the MIIT universities as a research subject is twofold. Firstly, within the similar social settings, there are great differences in embedded social and economic institutional mechanisms in different innovation systems, such as those involving knowledge and national defense ones. By taking the MIIT universities as a starting point, this research will describe the uniqueness of China’s national innovation system and provide insight into the effectiveness of this model in research publication compared with other universities in China. Secondly, in recent days, with the implementation of the Civil-Military Integration Development Strategy, the academic achievements of science and technology made by MIIT universities have been serving both the development of China’s national defense and the civil society. Therefore, the MIIT universities will play a more important role in the national innovation system. For these reasons, we will explore functions performed by the MIIT universities in the China’s national innovation system with the insight of Triple Helix model.

More recently, the mutual information methodology, a Triple Helix indicator, has been used to examine the extent to which networks of relations among university, industry and government have developed into a synergetic configuration at the national level[9] in countries including South Korea [16, 17], Japan [18, 19], and Sweden [20]. This method has also been used to analyze innovation systems at the national level, including sectoral, technological and regional innovation systems [8]. This research will take universities as a unit of analysis and compare the performance of UIG interaction in China.

## Background: National innovation system in China and MIIT universities

### National innovation system in China

In China, the basic unit for developing innovation systems is the province-level regions [21, 22] until the end of the last century. Since 1998, China began to adjust university administration system in order to reduce conflicts between central and local governments. The result is the establishment of two-level management of central and local governments. Universities under the management of the first level are called national universities, and they mainly serve national priorities with research-oriented and elite education. Other universities at the provincial level are known as regional universities. The main role of the regional higher education is to serve the regional development by providing mass higher education. At the national level, not all higher education institutions are administered by the Ministry of Education (MoE). In fact, some institutions are affiliated with other functional departments of the central government, including the Ministry of Public Security, the Ministry of Transport and the Ministry of Foreign Affairs and so on.

Underpinned by the Reform and Opening Up Policy, China has achieved economic prosperity and can be expected to become an innovation country. As described in the “Medium-to-Long-term Plan outline for the Development of National Science and Technology” (2006–2020) (MLP), the National Innovation System (NIS) includes four main sub-systems: the technological innovation system, the knowledge innovation system, the national defense innovation system, and the regional innovation system. Walsh and Francis (2011) have reported the great effects of the national defense innovation system on the advancement of China’s national innovative capacity [23]. According to the Triple Helix model, the MIIT universities play an important role in the national defense innovation system by focusing on cultivating professional talents and providing the theoretical basis and research approach for the national defense industry,

### Knowledge innovation system of seven MIIT universities at different historical stages

Recent decades have witnessed China’s rapid modernization of national defense and military. Quite a number of huge achievements in this field are, to a large extent, associated with universities, especially the MIIT universities. MIIT universities are seven universities affiliated to China’s Ministry of Industry and Information Technology, including Beihang University (BUAA), Beijing Institute of Technology (BIT), Harbin Institute of Technology (HIT), Harbin Engineering University (HEU), Nanjing University of Aeronautics and Astronautics (NUAA), Nanjing University of Science and Technology (NUST) and Northwestern Polytechnical University (NPU). These seven universities were managed by different departments of central government during their early years, and were later unified under the management of the Commission of Science, Technology and Industry for National Defense (CSTIND). In March 2008, the first session of the eleventh National People's Congress adopted the institutional restructuring of the State Council. Following this trend, the CSTIND was revoked and these seven universities became affiliated with MIIT. Despite belonging to different ministries in history, these seven universities have been contributing to national defense industry with professional talents and high-level technology. Therefore, the development of MIIT universities, to a large extent, was closely linked to the evolution of knowledge innovation system in China at different historical stages.

#### Between 1949 and 1978

After the foundation of People’s Republic of China in 1949, the government was faced with severe challenges of rebuilding the motherland. In order to establish the modern industrial system and cultivate practical experts, central government took steps to change original Soviet-modeled educational system. Therefore, a range of universities affiliated with government departments with industrial backgrounds were built and called industrial universities, including agricultural, forestry, and metallurgy universities. These universities have long been dependent on the development of their affiliated industry, cultivating specialized talents and inventing specific techniques for certain industries. As national defense industrial universities, MIIT universities play unique roles as the key institutions and are administered by different ministries. HIT was administered by the Ministry of Aviation; BIT and NUST were administered by China Weapon Industry Company; BUAA, NUAA and NPU were managed by the Ministry of Aviation; HEU was managed by the State Council and Central Military Commission.

The Cultural Revolution from 1966 to 1976 shocked Chinese educational system. The university entrance exams were cancelled after 1966, and many intellectuals including teachers and students were sent to rural areas. At this stage, universities’ daily routine was interrupted and so developing function made it impossible for MIIT universities.

#### Between 1978 and 2000

With the end of the Cultural Revolution, the university entrance exam was resumed in 1978. Meanwhile, central government began to rebuild higher education, and 88 universities, including all seven MIIT universities, were prioritized.

Along with the implementation of reform and opening-up policy, China began to shift from traditionally planned economy to market economy. The economic basis determines superstructure, and economic management system was expected to adapt to this trend. Since the mid-1980s, a comprehensive programme of institutional reforms was adopted at the level of the central government. Original higher education system (universities administered by relevant industry sectors) was adjusted accordingly. From 1998 to 2000, three institutional restructurings of higher education had been completed. All MIIT universities had been put under the administration of CSTIND, and expected to serve for the development of national defense.

#### After 2000

At the end of the 20th century, along with the new science and technology revolution, the world entered into the era of knowledge economy. The profound change has turned innovation into the driving force of social and economic development, as well as the major element among national competitions. The increasing demand for innovative talents and technologies have promoted the reform of higher education.

At the beginning of the 21^st^ century, MIIT universities began to expand their education scale and improve their operating system. The first major attempt for the MIIT universities was to cultivate professional compound talents. Rather than focusing on the skilled labor for related industries, MIIT universities have gradually transformed from a single-subject into comprehensive ones and developed a tendency to provide all-round and high quality talents for society. Besides, the seven MIIT universities struggle with their ability to improve their research level, not only in the industrial areas that they faced traditionally, but also in the cutting-edge science and technology fields, covering biomedicine, artificial intelligence, advanced materials, and integrated photovoltaics. These seven universities also targeted the development strategy of civil-military integration by applying technological research achievements into military equipment and commercialized products.

**Fig 1:**
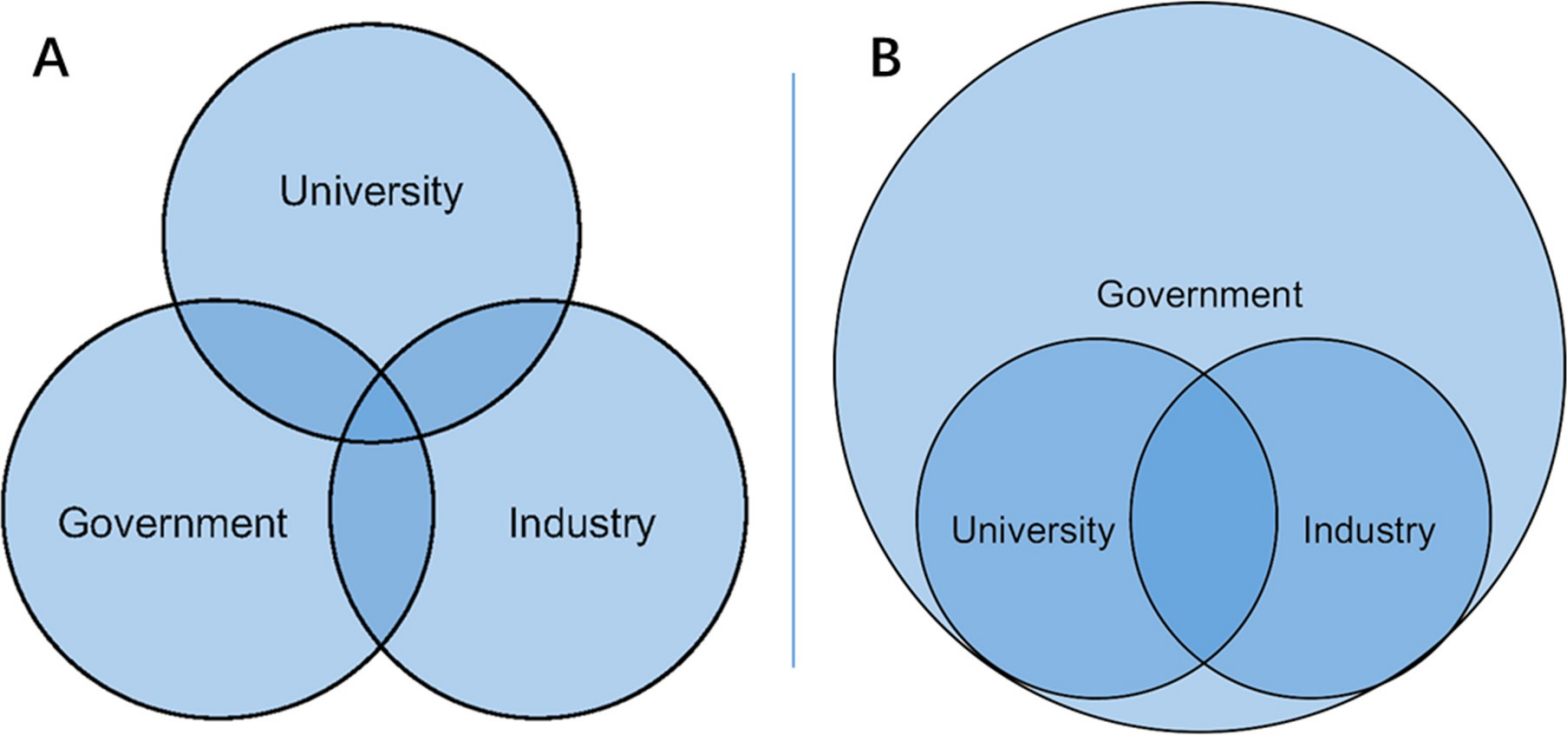
The triple helix model. A) The statist model B) The overlapping model.

From above analysis, MIIT universities have undergone different development stages. Before 1978, China was in the planning economic system, and in this context, the evolution of seven MIIT universities, including establishment, mergence and disassembly, was mainly government-pushed. Therefore, from the perspective of the Triple Helix model, the government plays the dominant role in the relationship between university, government and industry, while industries and universities are controlled by the state, namely the statist model [4, 9] (Fig 1A).

After the reform and opening-up in 1978, higher education is expected to cultivate talents and produce scientific achievements for market economy development. Especially in the era of knowledge economy, beyond teaching and research functions, MIIT universities tend to shoulder more public service functions, taking the initiative to foster students with creativity and produce scientific and technological achievements with original innovation capacity, targeting strategic opportunities and technology frontiers. Chinese universities, particularly the MIIT universities, tend to be entrepreneurial and gradually interacted with government and industry, namely the triple helix model [4, 9] (Fig 1B).

## Methods and materials: Measuring triple helix dynamics

### Methods

Measurement of the triple helix dynamics is based on the concept of entropy, which was first introduced by Boltzmann and Shannon’s information theory [24]. The entropy can be used to measure the uncertainty and disorder that exists in a system [25, 26]. When variation is considered as a relative frequency or probability distribution(∑_*i*_*p*_*i*_), the uncertainty contained in the distribution(*H*_*i*_) is defined as follows [24]:

$$H_{i}=-\sum_{i}p_{i}\;\log_{2}\left(p_{i}\right)$$ (1)

The number of publications from database on the Internet is *f*_*i*_, and its frequency is *p*_*i*_ = *f*_*i*_/∑_*i*_*f*_*i*_. The value *H*_*i*_ represents the average amount of information. Equivalently, two-dimensional entropy (*H*_*ij*_) is calculated as:

$$H_{ij}=-\sum_{i}\sum_{j}p_{ij}\;\log_{2}\left(p_{ij}\right)$$ (2)

The two-dimensional entropy (*H*_*ij*_) is the sum of the uncertainty in the two dimensions diminished with their mutual information. In other words, the two variations overlap in their co-variation and condition each other in the remaining variations.

Then, mutual information between two dimensions of the probability distribution is equal to the transmission(*T*_*ij*_) of the uncertainty [27, 28], which can be given as follows:

$$H_{ij}=H_{i}+H_{j}-T_{ij}$$ (3)

$$T_{ij}=H_{i}+H_{j}-H_{ij}$$ (4)

*T*_*ij*_ is zero if the two distributions are completely independent(i.e., the co-variation is zero), but otherwise necessarily positive [27]. Triple helix (TH) extends to one more dimension, such that every author is considered to have a university(u), industry(i), or government(g) affiliation [29]. For the third hypothesis about the synergic effect of the TH relationship, the transmission (*T*_*uig*_) of uncertainty based on Shannon’s information theory can be derived as follows:

$$T_{uig}=H_{u}+H_{i}+H_{g}-H_{ui}-H_{ug}-H_{ig}+H_{uig}$$ (5)

The result is that indicator (*T*_*uig*_) provides Shannon-type information about the uncertainty that prevails in the mutual information networks, which depends on the interaction among three sectors. Therefore, The *T*_*uig*_ value indicates “the size and the sign of the probabilistic entropy generated by the interaction within the complex system” is composed of three sub-dynamics: university, industry and government sectors [22, 28]. The indicator can be positive or negative (or zero). If the value for three-dimensional transmission (*T*_*uig*_) is negative, it indicates that the uncertainty prevailing at the network level is reduced and there exists a reciprocal synergy generated from collaboration among the university, industry and government sectors exists[28].

Note that the bilateral terms contribute to the reduction of uncertainty, while the three-dimensional uncertainty adds positively to the uncertainty which prevails at the network level.

### Data collection

The bibliometric data in this research came from the Web of Science (WoS) provided by the ISI of Clarivate Analytics, and the full records used in this paper were retrieved and downloaded on November 3, 2018. As the most authoritative database, the WoS can provide searching service for scientific publication that belong to the Science Citation Index (SCI), the Social Science Citation Index (SSCI), the Arts and Humanities Citation Index (A&HCI), and the Emerging Sources Citation Index (ESCI). As this study focuses on the MIIT universities and universities nationwide in China, data about bibliometric information of scientific papers written by authors from these addresses were collected from 2008 to 2017. After the data-cleaning process, the title and abstract of every record were read by our teammates to ensure that no invalid records were included. In order to search papers from China, the search query used was “AD = (China)”, meaning that the papers come from China (AD is the query for authors’ address). It shows that 2892991 papers from China was indexed by the WoS database in total. To search publications separately from universities, industry and government sectors and to categorize each other, we used “AD = (CHINA SAME (UNIV * OR COLL*))” standing for university(U), “AD = (CHINA SAME (GMBH* OR CORP* OR LTD* OR AG*))” standing for industry (I), and “AD = (CHINA SAME (NATL* OR NACL* OR NAZL* OR GOVT* OR MINIST* OR ACAD* OR NIH*))” standing for government (G). The publications of collaborations between “university and industry”, “university and government”, and “university industry and government” were labelled as “UI”, “UG”, and “UIG”, respectively [30].

In a similar way, datas of MIIT universities publication were collected. In order to compare seven MIIT universities further, the number of publications of each university is collected separately. For example, “OO = (Beihang Univ)” can be used when counting papers published by Beihang university,

## Research results

### Descriptive analysis

This paper firstly shows the basic situation of science and technology papers published by universities and their collaborators, including industries, governments and other universities. Table 1 provides the raw numbers of publication of MIIT and other Chinese universities based on the WoS database from 2008 to 2017.

**Table 1:** Publication of MIIT universities and universities in China based on the WoS database from 2008 to 2017.

	YEAR	U	UI	UG	UIG
MIIT UNIVERSITIES	2008	10587	195	983	39
	2009	11384	286	1708	52
	2010	12188	256	1539	46
	2011	13877	461	2298	75
	2012	16588	595	2862	116
	2013	18304	655	3400	122
	2014	21526	859	4002	158
	2015	23714	932	4691	179
	2016	27147	1171	5354	240
	2017	29103	1254	5876	300
UNIVERSITIES IN CHINA	2008	157629	7825	28322	2659
	2009	187563	10610	35187	3729
	2010	189743	11968	39364	4459
	2011	230910	16376	47688	5614
	2012	261332	19566	55777	6856
	2013	287630	21303	66514	8119
	2014	318248	23999	77298	9466
	2015	347457	27507	87218	10958
	2016	387337	32759	97243	13052
	2017	409175	35099	108137	14347

**Fig 2:**
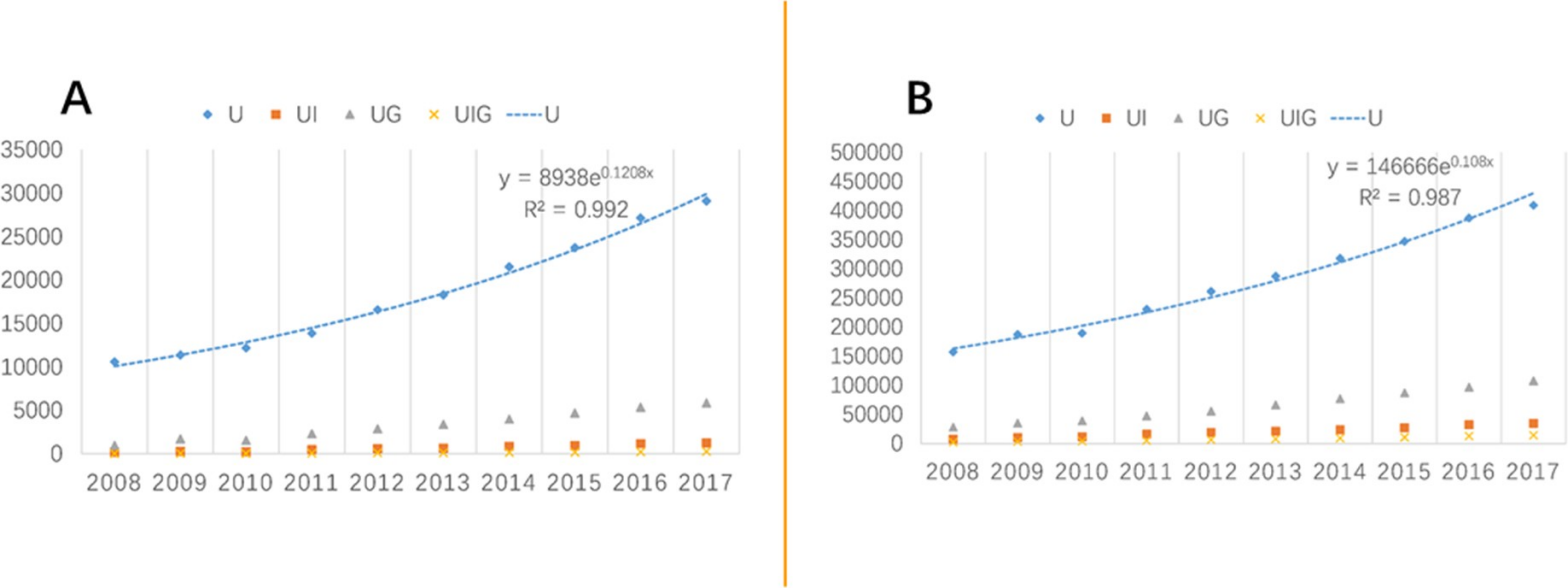
WoS publication. A)WoS publication by MIIT Universities. B)WoS publication by Universities in China.

The number of papers published by authors of MIIT universities and universities nationwide in China has increased steadily. In 2008, they produced 10587 and 157629 papers, respectively. By 2017, the number has grown to 29103 and 409175. Both MIIT universities and all universities in China have shown a consistent increase in papers in total. Further, the more detailed differences were revealed in Fig 2. From the specific collaboration with governments on publication (UG), MIIT universities have published scientific research papers from 983 to 5876 within ten years, with nearly 5-fold increase (Fig 2A). For universities nationwide, this number has risen from 28322 to 108137, with 2.8 times increase during the same period (Fig 2B). Then, papers published by MIIT universities and related industry sectors (UI) have increased from 195 in 2008 to 1254 in 2017, with 5.4 times increase. This figure has increased from 7825 to 35099 for the past ten years, increasing by 3.5 times. At last, the UIG publication of the MIIT universities and universities in China have increased by 7.7 times and 3 times from 2008 to 2017, respectively. From above analysis, although U, UG, UI and UIG publication for both MIIT universities and universities in China has increased between 2008 and 2017, the publication for MIIT universities has greater increases. In other words, MIIT universities, as a whole, publish more research papers than universities in China.

### Triple helix dynamics

**Fig 3:**
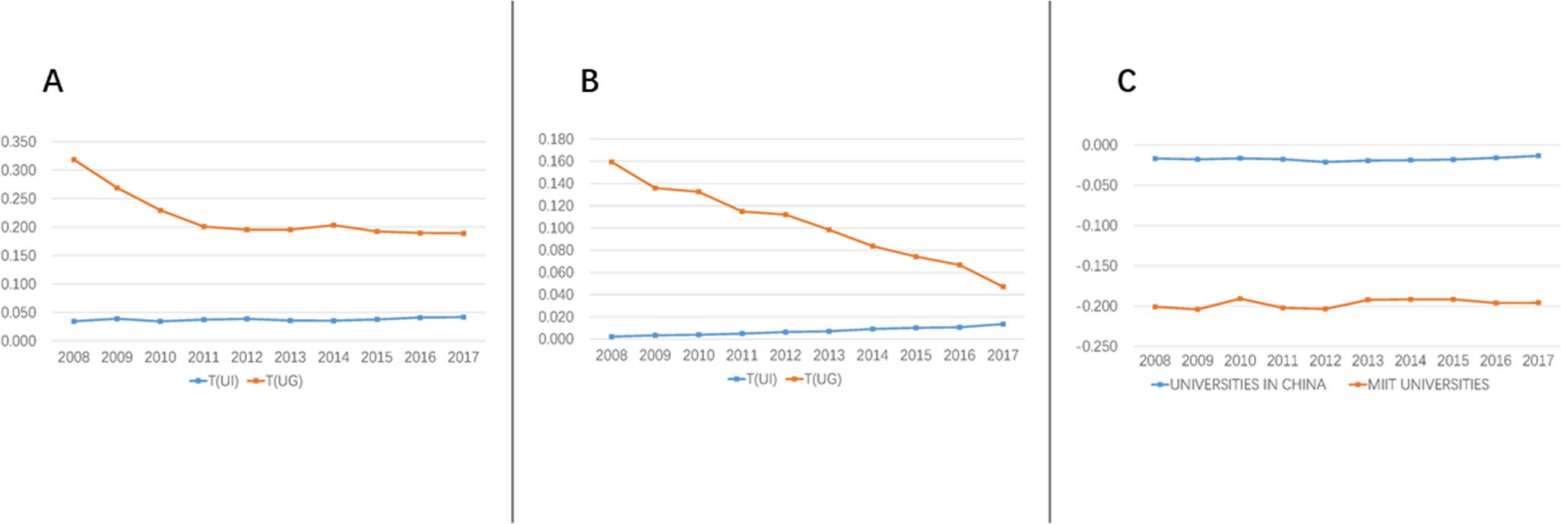
Transmission(T) of WoS by MIIT universities and universities nationwide in China. A) Transmission(T) of WoS in bilateral relations in MIIT universities. B) Transmission(T) of WoS in bilateral relations in universities in China. C) Transmission(T) of WoS on coordination among university, industry and government.

From these raw numbers, marginal probabilities (e.g. *P*_*u*_) can be derived, and from these probabilities, two- and three-dimensional transmission can be calculated. The results are presented in Table 2 and depicted graphically in Fig 3.

**Table 2:** Mutual information of scientific publications by MIIT universities and universities in China.

	YEAR	H(U)	H(I)	H(G)	H(UI)	H(UG)	H(IG)	H(UIG)	T(UI)	T(UG)	T(IG)	T(UIG)
**MIIT UNIVERSITIES**	2008	0.688	0.637	0.835	1.291	1.205	1.388	1.523	0.035	0.319	0.084	-0.201
	2009	0.677	0.690	0.839	1.328	1.247	1.434	1.599	0.039	0.269	0.094	-0.204
	2010	0.625	0.714	0.805	1.305	1.201	1.417	1.588	0.034	0.230	0.102	-0.191
	2011	0.630	0.762	0.836	1.354	1.265	1.474	1.663	0.038	0.201	0.124	-0.202
	2012	0.636	0.775	0.842	1.372	1.282	1.494	1.691	0.039	0.195	0.123	-0.203
	2013	0.629	0.761	0.819	1.354	1.252	1.468	1.672	0.036	0.196	0.112	-0.192
	2014	0.644	0.762	0.823	1.371	1.264	1.482	1.695	0.036	0.203	0.104	-0.191
	2015	0.643	0.775	0.825	1.380	1.275	1.494	1.715	0.038	0.192	0.105	-0.192
	2016	0.654	0.798	0.841	1.411	1.306	1.535	1.763	0.041	0.190	0.104	-0.196
	2017	0.658	0.799	0.840	1.415	1.310	1.539	1.771	0.042	0.189	0.100	-0.196
**UNIVERISITIES IN CHINA**	2008	0.420	0.303	0.804	0.720	1.064	1.103	1.345	0.002	0.159	0.003	-0.017
	2009	0.392	0.335	0.804	0.724	1.060	1.135	1.370	0.003	0.136	0.004	-0.018
	2010	0.403	0.363	0.836	0.762	1.106	1.194	1.444	0.004	0.132	0.005	-0.016
	2011	0.376	0.395	0.825	0.765	1.085	1.215	1.454	0.005	0.115	0.004	-0.018
	2012	0.389	0.414	0.836	0.797	1.114	1.246	1.496	0.006	0.112	0.004	-0.021
	2013	0.371	0.411	0.855	0.775	1.127	1.261	1.507	0.007	0.098	0.005	-0.019
	2014	0.351	0.420	0.863	0.761	1.130	1.278	1.517	0.009	0.084	0.005	-0.019
	2015	0.335	0.434	0.869	0.759	1.130	1.298	1.531	0.010	0.074	0.005	-0.018
	2016	0.314	0.452	0.864	0.755	1.111	1.310	1.531	0.011	0.067	0.006	-0.016
	2017	0.269	0.458	0.870	0.713	1.092	1.322	1.516	0.014	0.047	0.006	-0.013

Based on the information theory, in the two-dimension mutual information, the greater is the transmission (*T*_*ij*_), the closer are the related subjects. While in the three-dimension mutual information, the smaller transmission (*T*_*uig*_) indicates the greater synergistic effect of the Triple Helix relationship of university-government-industry. The transmission (*T*_*uig*_) of MIIT universities and universities nationwide in China is measured with Triple-helix quantitative algorithm.

**Table 2** shows the mutual information of scientific publications of MIIT universities and universities nationwide in China. Both MIIT universities and university nationwide show the T(UG)>T(UI), which means that MIIT universities and universities in China focus more on government collaboration (UG) than industry one (UI).

Further, an important insight into UG and UI collaboration among the bilateral relations for MIIT universities and universities in China is made. For MIIT universities (Fig 3A), the university -government research collaboration (*T*_*ug*_) has reduced sharply from 2008 to 2010. Since 2011, the *T*_*ug*_ value has remained stable, although this value increased slightly between 2013 and 2014; While the UI coordination for MIIT universities shows a rise between 2008 and 2017 in spite of the fluctuation. On the contrary, for universities nationwide in China (Fig 3B), the *T*_*ug*_ value has declined steadily and significantly since 2008, while the *T*_*ug*_ value has remained a slight rise.

Next, we examed the ultimate measure of *T*_*uig*_ (Fig 3C). Firstly, the *T*_*uig*_ value for both MIIT universities and all universities in China has been continuously negative from 2008 to 2017, which shows that the synergistic relationship of university-industry-government has been formed steadily. Secondly, the curve for the MIIT universities is under the national ones. This suggests that the MIIT universities have generated a greater synergistic effect of the Triple Helix relationship than universities in China. Thirdly, the *T*_*uig*_ for both MIIT universities and universities in China nearly keeps stable, and their values are close to zero, particularly for universities in China. This means that university-industry-government just shows initial synergy.

Above all, both MIIT universities and universities in China have more UG collaborations than UI ones. For the UG collaborations, the *T*_*ug*_ value of MIIT universities has remained stable, while that of universities in China has declined. For the UI collaborations, both MIIT universities and universities in China show steady growth. For the UIG collaborations, MIIT universities have a relatively greater synergistic effect of the Triple Helix relationship than university in China. For both MIIT universities and universities in China, the synergistic relationship of university-industry-government has been formed initially and steadily.

### Comparison of seven MIIT universities

**Fig 4:**
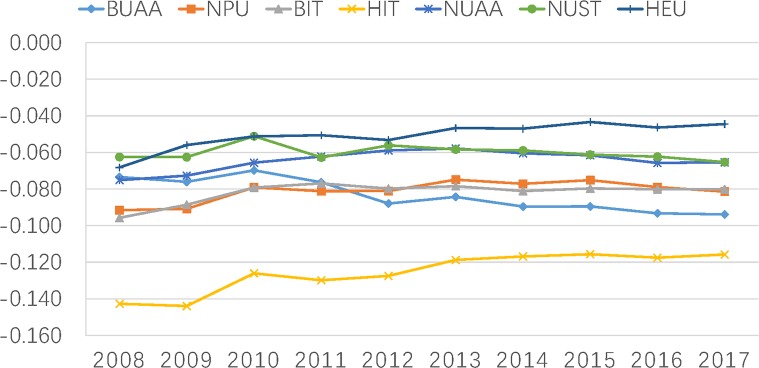
Transmission(T) of WoS by seven MIIT universities on coordination among university, industry and government.

Finally, let us contrast seven MIIT universities in more detail. Table 3 and Fig 4 first tell us that in the case of seven MIIT universities, the HIT has published the largest number of academic papers and has shown the most synergistic relations among university-government-industry since 2008. For the number of publications, there is litter difference among other six MIIT universities between 2008 and 2011. Since then, the BUAA, BIT and NPU have gradually published more papers than NUAA, NUST and HEU. In terms of *T*_*uig*_ for these other six MIIT universities, BUAA, BIT and NPU perform best in synergistic relations. Specially, BUAA has become much more synergistic with government and industry than other five MIIT universities since 2012. Unfortunately, the HIT showed less synergistic relations with other sectors between 2008 and 2013, and maintained steady until 2017.

**Table 3:** Publication of seven MIIT universities based on the WoS database from 2008 to 2017.

	BUAA	NPU	BIT	HIT	NUAA	NUST	HEU
2008	1062	1526	1661	3531	1062	820	925
2009	1343	1762	1720	4291	1193	1009	870
2010	1413	1631	1735	3747	1162	832	864
2011	1974	1972	1967	4362	1257	1374	971
2012	2773	2261	2468	5085	1385	1394	1222
2013	3147	2343	2914	5262	1594	1764	1280
2014	3947	2791	3416	5903	1972	1978	1519
2015	4474	3072	3624	6511	2187	2288	1558
2016	5156	3661	4014	7276	2627	2637	1776
2017	5612	4037	4277	7536	2752	2983	1906

## Discussion

As shown in Table 1 and Fig 2, both the MIIT universities and universities nationwide in China have increased their international academic publications for the past decade. As they are associated with policy direction of building world-class universities, MIIT universities and universities in China attach great importance to academic papers.

For more details, MIIT universities have published more papers than universities in China, and have been better synergistic with other sectors, including government and industry. To a large extent, this is the result of university administration system. As mentioned above, unlike most universities in China managed by the MoE, MIIT universities are administrated by the MIIT. Compared with MoE, MIIT has a closer relationship with industry and university, and plays the role as a bridge between them. Through setting up research program and projects, MIIT universities can published more papers and interact more with government and related industry.

According to Table 2, Fig 3A and Fig 3B, in terms of UG relations, the level of MIIT universities has remained stable in terms of UG relations, while the level of universities in China has declined. That is, why the UG relations show different trend? In China, most scientific research institutes were public sectors set up by government before 2000. The reform of government-funded public institution was put forth, and a trial of reforming institutions was carried out by type in 2000. In response to policy of reform, some institutes were transformed to enterprises, and the UG relations have shown a sharp decrease since 2008. On the other hand, based on national defense, MIIT universities mainly cooperate with scientific research institutes with national defense background. As these institutes play an important role in national security, they are still administrated by government. This may result in the more stable of UG relations of MIIT universities than other universities in China.

As can be seen in Table 2, Fig 3A and Fig 3B, both MIIT universities and universities in China have gradually developed relations with the government gradually (UI relations). In order to comprehensively promote the ability of innovation for economic and social development, central and local governments in China carry out a range of policies to support the cooperation between universities and industry. Especially the “2011 Plan” was particularly formulated and implemented to drive synergy innovation. With the guide of this plan, universities and industry in China interact with each other increasingly in the academic frontier and cutting-edge technologies.

As shown in Table 3 and Fig 4, in sight of seven MIIT universities, HIT, BUAA, BIT and NPU perform better. This result is related with the “Project 211” and “Project 985”. Universities are elected into these two projects can be financed by the central government to build as a world-class university. These two projects and other follow-up preferential policies enable elected universities to utilize policy and financial support to strengthen their research ability. Compared with “Project 211”, the “Project 985” can give the bigger financial support to elected universities. All the MIIT universities belongs to the “Project 211” universities. However, only HIT, BUAA, BIT and NPU are selected “Project 985”, which gives these four universities have an advantage over NUAA, NUST and HEU, and may lead to high publications and better synergistic with government and industry.

## Conclusions

In this paper, we focus on the innovation system of MIIT universities and universities in China in terms of university, government and industry coordination in scientific publications. The novelty of this paper lies in analysis of MIIT universities—one of Chinese-specific university, and measurement of their interaction with government and industry, based on the triple helix theoretical model and Shannon’s information entropy. The results are summarised as follows. Firstly, both the MIIT universities and universities nationwide in China have increased their international academic publications for the past decade, and publications of MIIT universities increase greater, not only in total publications, but also in UG, UI and UIG publications. Secondly, in terms of U-I-G interaction, For UG relations, the *T*_*ug*_ value of MIIT universities has remained stable, while that of universities in China has declined. For UI relations, the *T*_*ug*_ values of both MIIT universities and universities in China show steady growth. For UIG relations, MIIT universities have a relatively greater synergic effect of the TH relationship than university in China. Thirdly, for more details in seven MIIT universities, the BUAA, BIT and NPU have published more papers, and have been more synergistic with government and industry than NUAA, NUST and HEU.

More efforts are needed to improve U-I-G interaction. First of all, policy incentives for universities play an important role in synergy of U-I-G relations. For example, the “Project 211”, “Project 985” and “2011 Plan” increase paper publications and strengthen U-I-G relations. Next, reasonable administrative system according to national conditions, can be in favour of paper publication. Further exploration of the role of governments in national innovation system and U-I-G interaction mode is needed. Moreover, as the synergy of U-I-G relations is formed initially in China, universities and other social sectors should strengthen coordination, and pay more attention to design reasonable U-I-G interaction mode.
